# Study on Adsorption Characteristics and Water Retention Properties of Attapulgite–Sodium Polyacrylate and Polyacrylamide to Trace Metal Cadmium Ion

**DOI:** 10.3390/polym16121756

**Published:** 2024-06-20

**Authors:** Ziming Cai, Feng Zhan, Yingnan Wang, Meiling Wu, Lingjian Kong, An Wang, Zhanbin Huang

**Affiliations:** 1 School of Resources, Environment and Materials, Guangxi University, Nanning 530004, China; 2015391002@st.gxu.edu.cn; 2 School of Chemical and Environmental Engineering, China University of Mining and Technology (Beijing), Beijing 100083, China; wangyingsouth@163.com (Y.W.); zqt2100302085@cumtb.edu.cn (M.W.); ljkong2023@163.com (L.K.); wanganchina@126.com (A.W.)

**Keywords:** superabsorbent polymer, cadmium ion (Cd(II)), adsorption capacity, adsorption kinetics, water adsorption ratio

## Abstract

The adsorption mechanism of superabsorbent polymer (SAP) can provide theoretical guidance for their practical applications in different environments. However, there has been limited research on the mechanism of attapulgite–sodium polyacrylate. This research aimed to compare the Cd(II) adsorption characteristics and water retention properties of organic–inorganic composite SAP (attapulgite–sodium polyacrylate, OSAP) and organic SAP (polyacrylamide, JSAP). Batch experiments were used to investigate the kinetics of Cd(II) adsorption, as well as the thermodynamic properties and factors influencing these properties. The results show that the Cd(II) adsorption capacity was directly proportional to the pH value. The maximum adsorption capacities of OSAP and JSAP were of 770 and 345 mg·g^−1^. The Cd(II) adsorption for OSAP and JSAP conformed to the Langmuir and the quasi-second-order kinetic model. This indicates that chemical adsorption is the primary mechanism. The adsorption process was endothermic (ΔH_0_ > 0) and spontaneous (ΔG_0_ < 0). The water adsorption ratios of OSAP and SAP were 474.8 and 152.6 in pure water. The ratio decreases with the increase in Cd(II) concentration. OSAP and JSAP retained 67.23% and 38.37% of the initial water adsorption after six iterations of water adsorption. Hence, OSAP is more suitable than JSAP for agricultural and environmental ecological restoration in arid and semi-arid regions.

## 1. Introduction

Trace metal pollution has emerged as a paramount environmental concern. The management of trace metals has garnered significant interest due to their enduring presence in the environment and resistance to degradation [1]. Cadmium Cd(II), an exceptionally hazardous trace metal, is predominantly sourced from the mining and metallurgical sectors, as well as the manufacturing of nickel–cadmium batteries, pigments, and plastic stabilizers. It also enters the environment via atmospheric diffusion, as well as solid and water migration, ultimately contributing to soil contamination. Cadmium exposure has been associated with the development of prostate, lung, and testicular cancer in the human population [2]. In soil and aqueous solutions, Cd typically occurs in the form of divalent Cd(II) and predominantly flows under aerobic, acidic conditions [3]. Current techniques for removing Cd(II) from soil consist of bioremediation, agronomic methods, and physical and chemical passivation [4]. Passivation technology is widely utilized in the majority of restoration projects due to its low secondary contamination, simple operation, and high process efficiency, making it one of the most prevalent technologies [5]. The application and research of SAP have been ongoing for 40 years, and it is a vital resource for drought resistance, water conservation, soil enhancement, and fertility preservation for China’s agricultural production. In recent years, with the growing attention to the remediation of soil trace metal pollution, the use of SAP for removing soil trace metal pollution has attracted significant interest [6,7]. However, there is a lack of research on the adsorption and antagonism of soil trace metal ions toward different types of SAPs in this field, which provides an important foundation for this study.

The SAP is a complex molecular structure polymer compound. Hydrophilic functional groups, including carboxyl and hydroxyl groups, have the ability to adsorb and exchange cations in soil. This characteristic is beneficial for building soil structure and retaining water and fertilizers. SAP has broad application prospects in water-saving agriculture and environmental management [8]. SAPs can be classified into organic, organic-inorganic composite, and inorganic types from the perspective of chemical composition. JSAP is a highly water-retentive organic SAP, characterized by a cross-linked molecular structure [9]. Natural clay minerals are one of the main components of organic–inorganic composite OSAP. Attapulgite are easily accessible and cost-effective. The synthesis method of OSAP is relatively simple and straightforward. So far, there has been limited research on the adsorption mechanisms of OSAP and JSAP in the field of materials. Practical engineering dosages of SAP are determined primarily through experience, lacking a reliable quantitative standard. The physical and chemical properties of different SAPs vary significantly due to the use of different materials and production processes in production. Therefore, the effectiveness of these SAPs in removing trace metal from the environment also varies in practice. This hampers the rational selection of SAPs and, to some extent, constrains their application. Zhang, Y. et al. studied the impact of SAP synthesized from acrylic acid, acrylamide, and N,N′-methylene bisacrylamide on the accumulation of Cd in plants and soil quality [4]. Dhiman, J. et al. examined the effects of Plantago major peel biochar, and polyacrylamide SAP on the accumulation of trace metals in potato, spinach, and soil [5,6,7]. Li, J. C. et al. studied the preparation of magnetic Fe_3_O_4_/ZIF-8 (MFZ) and its adsorption performance for Cd(II) in water. The maximum saturation adsorption capacity is 160.26 mg·g^−1^, and the cost of treating wastewater with a Cd(II) concentration of 40 mg·L^−1^ is of approximately USD 8.35 per cubic meter [8]. Wang, P. studied the adsorption mechanism of Cd(II) using coal-derived humic acid modified with Ca(OH)_2_ [9]. Han, X. et al. explored the adsorption mechanism of Cd(II) using a hydrothermal carbonization-derived hydrochar [10]. Han, X. et al. examined the adsorption mechanism of Cd(II) using a hydrochar prepared by hydrothermal carbonization, with a maximum adsorption capacity of 24.53 mg·g^−1^ for Cd(II) [10]. Cai, Y. R. et al. studied the adsorption mechanisms of three adsorbents. The maximum adsorption capacities of the three adsorbents are of 10.20 mg·g^−1^, 39.99 mg·g^−1^, and 48.82 mg·g^−1^ for Cd(II), respectively [11].

This study examined the influence of Cd(II) on the adsorption characteristics of OSAP and JSAP. This study of the adsorption mechanisms of OSAP and JSAP, representing hybrid organic–inorganic and organic SAP, respectively, contributes to a better selection and application of OSAP and JSAP, as well as the formulation of corresponding application strategies. We explored the adsorption mechanism of Cd(II) through analysis and characterization. We compared the differences between OSAP and JSAP through batch experiments and investigated the effect of Cd(II) on water retention. Overall, our study fills the knowledge gap regarding OSAP and JSAP, providing a significant contribution to the advancement of the field and offering theoretical foundations and guidance for practical applications in related areas.

## 2. Material and Methods

### 2.1. Preparation of Superabsorbent Polymers

**Figure 1:**
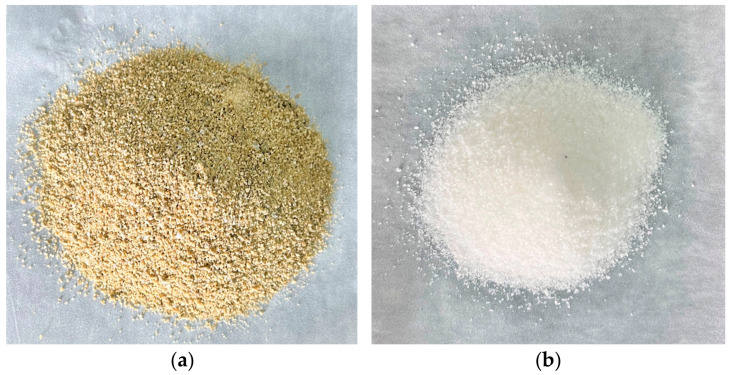
Superabsorbent polymers. (**a**) OSAP; (**b**) JSAP.

The attapulgite–sodium polyacrylate (OSAP) was from Lanzhou Institute of Chemical Sciences, Chinese Academy of Sciences, and manufactured by Changan Industrial Group Co., Ltd., Shengli Oilfield (Dongying, Shandong, China). Polyacrylamide (JSAP) is a French Essen superabsorbent polymer supplied by Beijing Jinyuanyi Ecological Engineering Technology Center (Beijing, China) for Agriculture and Forestry. Figure 1 shows the real image of the SAPs.

### 2.2. Batch Experiments

#### 2.2.1. Dosage Experiment

The CdCl_2_·4H_2_O (Anhui ZeSheng Technology Co., Ltd., Anqing, China) solution was prepared using pure water as the background solution. Different dosages (10 mg, 20 mg, 30 mg, 40 mg, 50 mg, and 60 mg) of OSAP and JSAP were separately mixed with 50 mL Cd(II) solutions at 200 mg∙L^−1^ in centrifuge tubes. The pH was adjusted to 5.0 using 0.1 mol·L^−1^ HNO_3_ (Beijing Lanyi Chemical Products Co., Ltd., Beijing, China) or NaOH (Lanyi Chemical Products Co., Ltd., Beijing, China). The centrifuge tubes were placed on an oscillator and shaken for 24 h at 25 °C to reach reaction equilibrium. The oscillation rate was of 200 rpm. The solution was filtered using a membrane filter with a pore size of 0.45 μm. The content of Cd(II) in the filtered solution was determined by ICP-OES analysis. 

The iCAP™ 7400 Inductively Coupled Plasma-Atomic Emission Spectrometer (ICP-OES) (Thermo Fisher Scientific, Waltham, MA, USA) was used for detecting the Cd(II) ion concentration of the filtered solution after filtration through a 0.45 µm filter membrane. The SQP Electronic balance (Sartorius Scientific Instruments Co., Ltd., Gottingen, Lower Saxony, Germany) was used for sample weighing. The FE20K pH meter (Mettler Toledo International Inc., Zurich, Switzerland) was used for pH measurement. The HZQ-F100 constant temperature oscillating incubator (Suzhou Pei Ying experimental Instrument Co., Ltd., Suzhou, China) was used to maintain constant temperature and vibration.

#### 2.2.2. Cd(II) Concentration Experiment 

A total of 30 mg of OSAP and 40 mg of JSAP were separately mixed with 50 mL Cd(II) solutions at different concentrations (50, 100, 150, 200, 250, and 300 mg∙L^−1^) in centrifuge tubes. The pH was adjusted to 5.0 using 0.1 mol∙L^−1^ HNO_3_ or NaOH. The centrifuge tubes were placed on an oscillator and shaken for 24 h at 25 °C at 200 rpm. The method for filtering the solution and measuring the concentration was the same as the method described above.

#### 2.2.3. Adsorption pH Experiment 

A total of 30 mg of OSAP and 40 mg of JSAP were separately mixed with 50 mL Cd(II) solutions at 250 mg∙L^−1^ concentration in centrifuge tubes. The pH was adjusted to different values (2, 3, 4, 5, and 6) using 0.01 mol∙L^−1^ HNO_3_ or 0.01 mol∙L^−1^ NaOH. The centrifuge tubes were placed on an oscillator and shaken for 24 h at 25 °C. The method for filtering the solution and measuring the concentration was the same as the method described above.

#### 2.2.4. Temperature Experiment 

A total of 30 mg of OSAP and 40 mg of JSAP were separately mixed with 50 mL Cd(II) solutions at 250 mg∙L^−1^ concentration in centrifuge tubes. The pH was adjusted to 5.0 using 0.1 mol∙L^−1^ HNO_3_ or NaOH. The centrifuge tubes were placed on an oscillator and shaken for 24 h. The temperature of the oscillator was set to 15 °C, 25 °C, and 35 °C respectively, at 200 rpm. The method for filtering the solution and measuring the concentration was the same as the method described above.

#### 2.2.5. Desorption Experiment

A total of 30 mg of OSAP and 40 mg of JSAP were separately mixed with 50 mL Cd(II) solutions at 250 mg∙L^−1^ concentration in centrifuge tubes. The pH was adjusted to 5.0 using 0.1 mol∙L^−1^ HNO_3_ or NaOH. The centrifuge tubes were placed on an oscillator and shaken for 24 h at 25 °C. The solution was filtered using a membrane filter with a pore size of 0.45 μm. The OSAP/JSAP residues were dried for later use after being washed three times with pure water (pH = 5.0) to remove any unadsorbed Cd(II).

The OSAP/JSAP residues were separately mixed with pure water in centrifuge tubes. The pH was adjusted to different values (2, 3, 4, 5, and 6) using 0.01 mol∙L^−1^ HNO_3_ or 0.01 mol∙L^−1^ NaOH. The centrifuge tubes were placed on an oscillator and shaken for 24 h at 25 °C. The method for filtering the solution and measuring the concentration was the same as the method described above. See Text S4 for a detailed desorption quantity and desorption rate calculation.

#### 2.2.6. Adsorption Isotherms Experiment

This section utilizes the experimental data obtained from the temperature experiment described in Section 2.2.4, and the date is used to fit the experimental adsorption model.

We used the Freundlich isothermal model, Langmuir isothermal model, and Temkin model to fit the equilibrium adsorption data. See Text S2 for detailed model calculations.

#### 2.2.7. Kinetic Adsorption Experiment

A total of 30 mg of OSAP and 40 mg of JSAP were separately mixed with 500 mL Cd(II) solutions at 200 mg∙L^−1^ concentration in beaker. The pH was adjusted to 5.0 using 0.1 mol∙L^−1^ HNO_3_ or NaOH. Then, 5 mL samples were extracted from the solution using a syringe at reaction times of 5, 10, 20, 40, 80, 120, and 240 min. The method for filtering the samples and measuring the concentration was the same as the method described above.

The pseudo-first-order and pseudo-second-order kinetic models are used to fit the Cd(II) adsorption data for investigating the adsorption kinetics behavior of OSAP and JSAP on Cd(II) at different times [10]. See for Text S3 for detailed model calculations.

The DF-101S constant temperature magnetic stirring meter (Zhengzhou Great Wall Science and Trade Co., Ltd., Zhengzhou, China) was used to facilitate thorough adsorption reaction.

#### 2.2.8. Solid Surface Characterization Technique

We used Thermo escalab 250XI X-ray photoelectron spectroscopy (XPS) (Thermo Fisher Scientific, Waltham, MA, USA) to identify and analyze the chemical structure of substances. The relevant parameters of the XPS test machine are as follows: Monochromatic Al Kα source (hv = 1486.6 eV); Power: 150 W; Beam spot size: 650 μm; Voltage: 14.8 kV; Current: 1.6 A; Charge correction using contaminant carbon C 1s = 284.8 eV; Full spectrum transmission energy: 100 eV, step size: 1 eV; Narrow spectrum transmission energy: 20 eV, step size: 0.1 eV. The 20 mg powdered SAP was pressed into a 3 × 3 mm square shape with a height of 8 mm. The pressed surface needed to be kept clean and flat. The pressed sample was fixed on the sample stage, and the testing chamber was evacuated. The sample surface was irradiated with X-rays, and the photoelectron spectra were measured and recorded.

We used the Gemini300 thermal field emission scanning electron microscope (SEM) (Zeiss, Oberkochen, Baden-Wurtberg Oblast, Germany) to observe and analyze the composition, morphology, and structure of the samples on the surface. We adjusted the contrast value to set the noise parameter to 60. We adjusted the brightness value to achieve a grayscale parameter of around 20 on the screen. The filament heating was adjusted to between 6 and 7. The bias current was adjusted to around 100 microamps. We selected a magnification of 1000×.

We utilized the Nicolet 6700 Fourier Transform Infrared Spectroscope (FTIR) (Thermo Fisher Scientific, Waltham, MA, USA) to analyze the functional groups of the samples. The FTIR test machine operated within a wavenumber range of 400–4000 cm^−1^. The spectrometer boasted a resolution of 4 cm^−1^, a signal-to-noise ratio of 50,000:1, and conducted 32 scans. Then, 20 mg powdered SAP was pressed into a 3 × 3 mm square shape. We measured the baseline to calibrate the instrument to compensate for environmental factors before recording the sample spectra. Infrared light ranging from 500 to 4000 wavenumbers was used to scan the sample.

#### 2.2.9. Water Adsorption Experiment

A total of 30 mg of OSAP and 40 mg of JSAP were separately mixed with pure water and Cd(II) solutions at different concentrations (100, 150, 200, 250, and 300 mg∙L^−1^) in beakers. The pH was adjusted to 5.0 using 0.1 mol∙L^−1^ HNO_3_ or NaOH. The beakers were placed on an oscillator and shaken for 24 h at 25 °C at 200 rpm. Then, they were removed from the water and weighed to calculate the water adsorption ratio. Finally, they were left to air for natural dehydration, and weighed at 0, 15, 30, 45, 60, 75, 90, and 105 min to measure their water-retaining property. The operation above was repeated six times to measure the repeated water adsorption property.

## 3. Results and Discussion

### 3.1. Effect of Different Factors on Cd(II) Adsorption Characteristic of OSAP and JSAP

#### 3.1.1. Effect of Different Dosages on Cd(II) Adsorption

**Figure 2:**
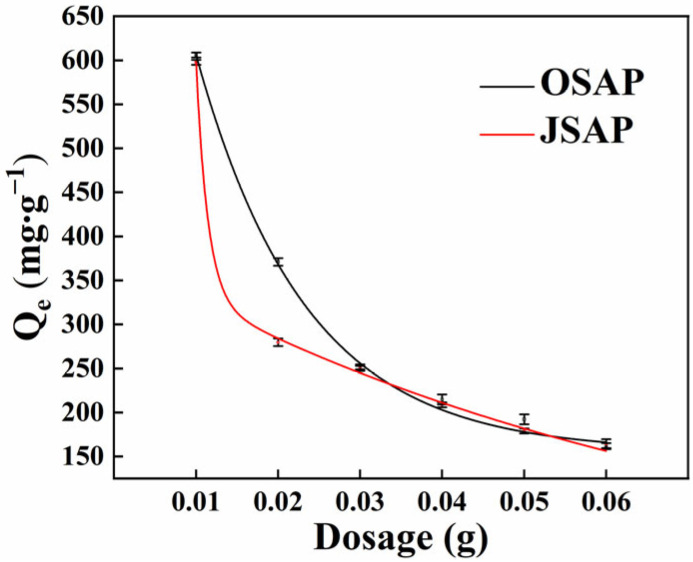
Cd(II) adsorption capacities by SAP in different dosages.

The SAPs with different dosages were used to adsorb Cd(II) to determine the optimal dosage of OSAP and JSAP. As shown in Figure 2, when the dosage of OSAP/JSAP was in the range of 0.01–0.03 g, the amount of Cd(II) adsorbed initially decreased rapidly, followed by a slower decline. The inflection point occurred at 0.03 g. The inflection points for OSAP and JSAP occurred at 0.03 g and 0.02 g, respectively. We chose the dosage of one unit to the right of the inflection point as the best dosage of the SAP. $Q_{e}$ is the adsorption capacity of Cd(II). The calculation method of $Q_{e}$ is detailed in Text S1.

#### 3.1.2. Effect of Cd(II) Concentration on Adsorption 

**Figure 3:**
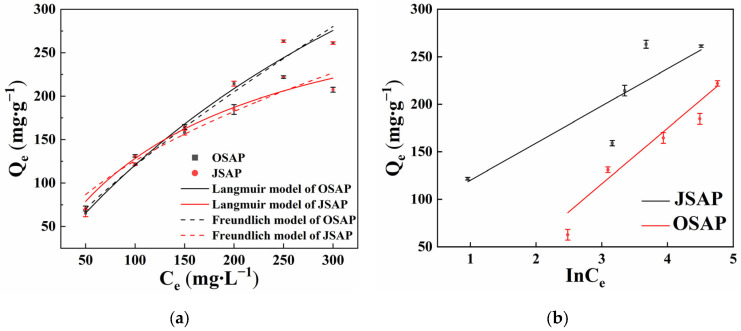
Cd(II) adsorption capacities by SAP in different initial Cd(II) concentrations. (**a**) Langmuir and Freundlich model; (**b**) Temkin model.

The Langmuir and Freundlich adsorption models were used to fit the adsorption process of Cd(II) by OSAP/JSAP [11]. As shown in Figure 3a, the amount of adsorbed Cd(II) increases with increasing solution concentration. Figure 3b illustrates the fitting of the adsorption process with the Temkin model. C_e_ is the residual concentration of Cd(II) in the solution. InC_e_ means “taking the logarithm of C_e_”.

Table 1 shows the fitting parameter. The results show that the Langmuir model was suitable for OSAP, as follows: Langmuir (R^2^ = 0.977) > Freundlich (R^2^ = 0.970) > Temkin (R^2^ = 0.774). For JSAP, the Langmuir model was suitable, as follows: Langmuir (R^2^ = 0.966) > Temkin (R^2^ = 0.949) > Freundlich (R^2^ = 0.935). This indicates that the Langmuir model can better describe the adsorption behavior of OSAP/JSAP [12]. The maximum adsorption capacities of Cd(II) by OSAP/JSAP were of 770 mg·g^−1^ and 345 mg·g^−1^, respectively. OSAP has a higher adsorption ratio for Cd(II) than JSAP, and the fitting with the Langmuir model indicates that Cd(II) covered the surface sites of OSAP/JSAP in a monolayer [13]. Consequently, the mode of Cd(II) adsorption on the material’s surface is a single-layer adsorption.

#### 3.1.3. Effect of pH on Cd(II) Adsorption

**Figure 4:**
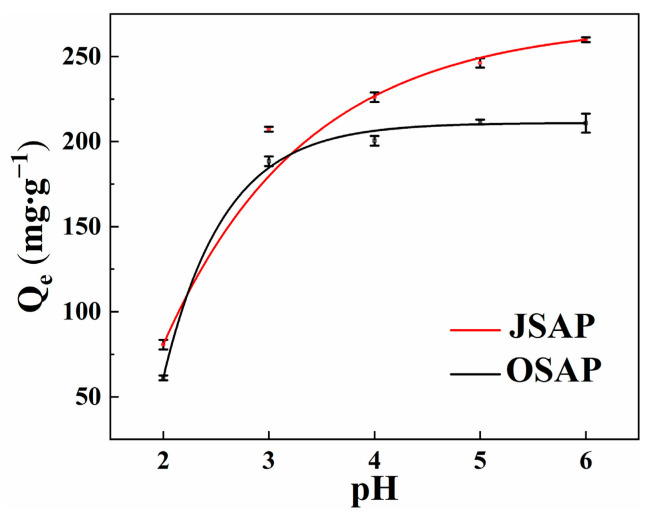
Cd(II) adsorption capacities by SAP at different pH levels.

The pH value significantly affects the adsorption capacity of trace metals. It is an important parameter in adsorption research as it reflects the surface charge of the adsorbent [14,15]. Cd(II) in the solution begins to precipitate and convert into Cd(OH)_2_ when the pH value exceeds 7. Therefore, experiments on the adsorption capacity for Cd(II) are not conducted when the pH value exceeds 7. Figure 4 shows the Cd(II) adsorption capacity by OSAP/JSAP at different pH levels. Cd(II) adsorption capacity increases significantly within the initial pH range of 2.0–3.0. The adsorption capacity for Cd(II) is low at lower pH values. The reason for this is that the increased concentration of H^+^ in the solution leads to competition between cations. Cd(II) does not bind with surface functional groups, leading to lower adsorption capacity of OSAP/JSAP for Cd(II) at low pH values. A series of acidic functional groups undergo dissociation when the pH value rises. The electrostatic interaction between negatively charged functional groups and Cd(II) enhances the adsorption capacity [16].

The pH of acidic soil generally ranges from 4 to 6. There is a significant decrease in adsorption capacity in the range of 2–3, while the decrease is not significant between 3 and 7. Additionally, in arid to semi-arid regions, SAP can provide moisture while also adsorbing trace metal, demonstrating excellent economic viability.

#### 3.1.4. Effect of Temperature on Cd(II) Adsorption

**Figure 5:**
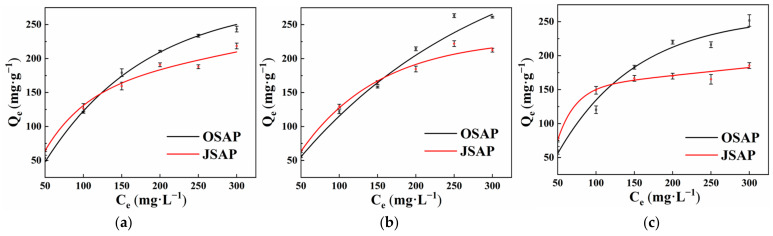
Effect of different temperatures 15° (**a**), 25° (**b**), 35° (**c**) on the adsorption of Cd(II).

Temperature is an important factor affecting the adsorption of trace metal. Increasing the temperature can increase the reaction rate and promote the adsorption of trace metals on the adsorbent. Figure 5a–c shows the effect of different temperatures, namely 15°, 25°, and 35°, on the adsorption of Cd(II). The results illustrate that the adsorption capacity of Cd(II) for OSPA increased from 53.36 mg·g^−1^ to 60.58 mg·g^−1^ when the temperature increased from 15 °C to 35 °C. For JSAP, the adsorption capacity of Cd(II) increased from 64.43 mg·g^−1^ to 75.82 mg·g^−1^. OSAP and JSAP improved by 7.22% and 11.39%, respectively. Temperature has a significant impact on enhancing the adsorption capacity for Cd(II). The adsorption capacity of Cd(II) is lowest at 15 °C and highest at 35 °C. This phenomenon may be explained by the following: Increasing temperature can speed up Cd(II) migration at the solid–liquid two-phase interface, increase Cd(II) movement rates in solutions, and enhance ion activity, which increases the likelihood of adsorption reactions occurring. An increase in temperature can enhance the activity of adsorption sites for Cd(II), which increases the amount of Cd(II) adsorbed.

### 3.2. Stability Analysis of Cd(II) Adsorbed by SAP

**Figure 6:**
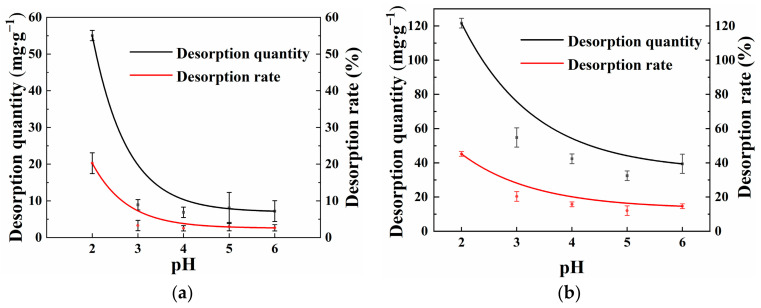
Cd(II) desorption capacity and desorption rate by (**a**) OSAP and (**b**) JSAP in different pH levels.

Studying the desorption characteristics of Cd(II) on OSAP/JSAP is crucial for determining adsorption stability. Figure 6a,b shows the effect of OSAP and JSAP on Cd(II) desorption at different pH levels. The results illustrate that the desorption amounts of OSAP/JSAP decrease sharply in the initial pH range of 2.0–3.0, and the desorption amounts slowly balance with the increase in pH from 3.0 to 6.0. The desorption rates of Cd(II) by OSAP and JSAP at a pH of 3 were of 3.28% and 20.37%, respectively. OSAP has lower desorption quantity, demonstrating greater stability compared to JSAP under different pH conditions.

### 3.3. Adsorption Modeling

#### 3.3.1. Adsorption Isotherm

**Figure 7:**
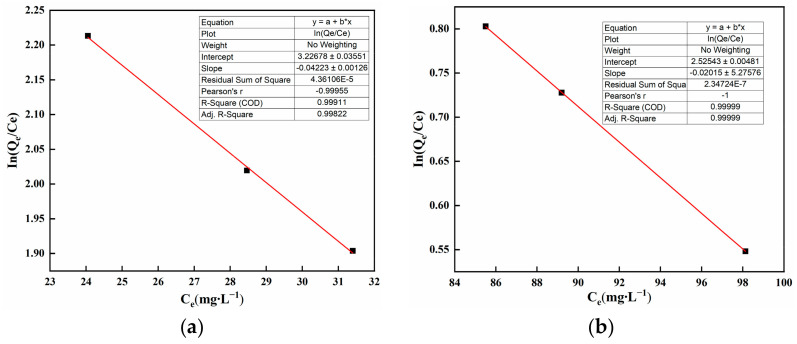
Thermodynamic parameter fitting graph.

As shown in Figure 7a,b, the horizontal axis $C_{e}$ is the residual Cd(II) concentration in solution, while the vertical axis is $In\left(\frac{Q_{e}}{C_{e}}\right)$. The slope is the equilibrium constant K. Both the slope and intercept are used for obtaining ΔG^0^. Subsequently, ΔG^0^ is used to calculate ΔH^0^ and ΔS^0^. R^2^ (R-squared), also known as the coefficient of determination, is an important concept in statistics used to measure the goodness of fit of a statistical model.

The thermodynamic parameters are shown in Table 2. With ∆G^0^ < 0 and ∆H^0^ > 0, it indicates that the adsorption process is spontaneous and endothermic. Furthermore, an increase in reaction temperature is conducive to the progression of the reaction. A positive value of ΔS^0^ indicates that there is an increase in the freedom of the solid–liquid interface during the adsorption process. An increase in freedom allows ions to interact with water molecules already adsorbed on the material surface, facilitating the adsorption reaction of SAP and Cd(II). See Text S5 for detailed ΔG^0^, ΔH^0^, and ΔS^0^ calculations.

#### 3.3.2. Adsorption Kinetics

**Figure 8:**
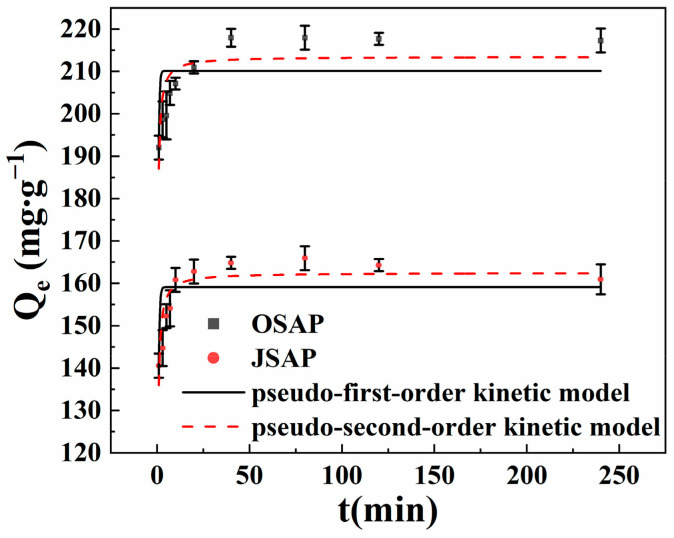
Cd(II) adsorption capacities by SAP in different contact times.

As shown in Figure 8, the adsorption capacity of Cd(II) rapidly increases within the initial 10 min of contact time, followed by a slow increase, and eventually reaches adsorption equilibrium after approximately 120 min. The adsorption sites on the surfaces of OSAP/JSAP facilitate the adsorption of Cd(II) onto the surface, which explains the reason for the rapid adsorption [17]. The adsorption rate of OSAP was also greater than that of JSAP. One possible explanation for this is that the larger pore size of OSAP can facilitate the migration of Cd(II), which increases the adsorption rate. With the reaction time increasing, Cd(II) occupied the majority of adsorption sites, indicating that intraparticle diffusion predominates until reaching equilibrium [18]. Table 3 shows the fitting parameters. The results indicate that the quasi-second-order model provides a more accurate description of the adsorption data in comparison to the quasi-first-order model. This indicates that chemical adsorption is the primary component of the adsorption mechanism for OSAP/JSAP [19,20].

### 3.4. Adsorption Mechanism by Material Surface Features

The primary factor in determining physical adsorption is the specific surface area of the material, which is positively correlated with van der Waals forces. An increase in the specific surface area of the material leads to enhanced van der Waals forces, resulting in an improvement in the adsorption capacity of the adsorbent [21]. Kinetic experiments indicate that the adsorption of Cd(II) by materials follows chemical adsorption and is characterized by quasi-second-order kinetic models [22]; Materials with greater specific surface areas, such as zeolite [23] and biochar [24], have a greater specific surface area than the material in question. 

#### 3.4.1. XPS Analysis 

**Figure 9:**
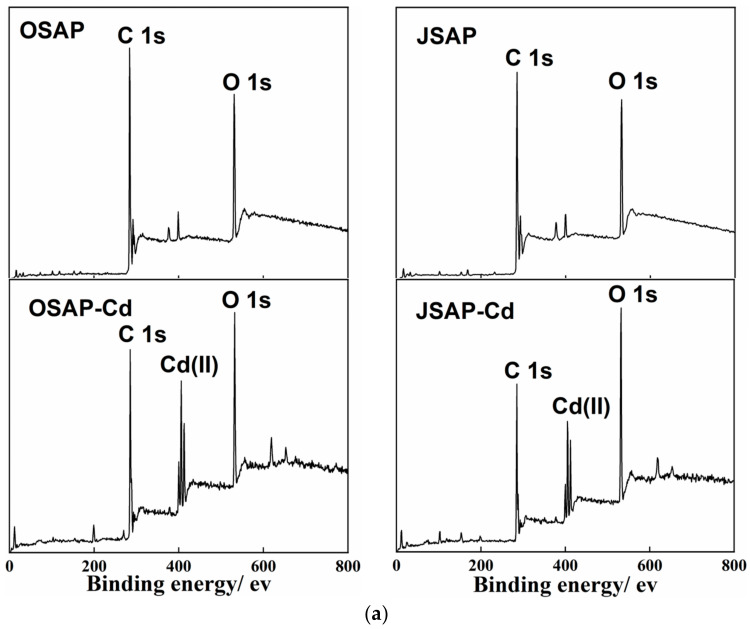

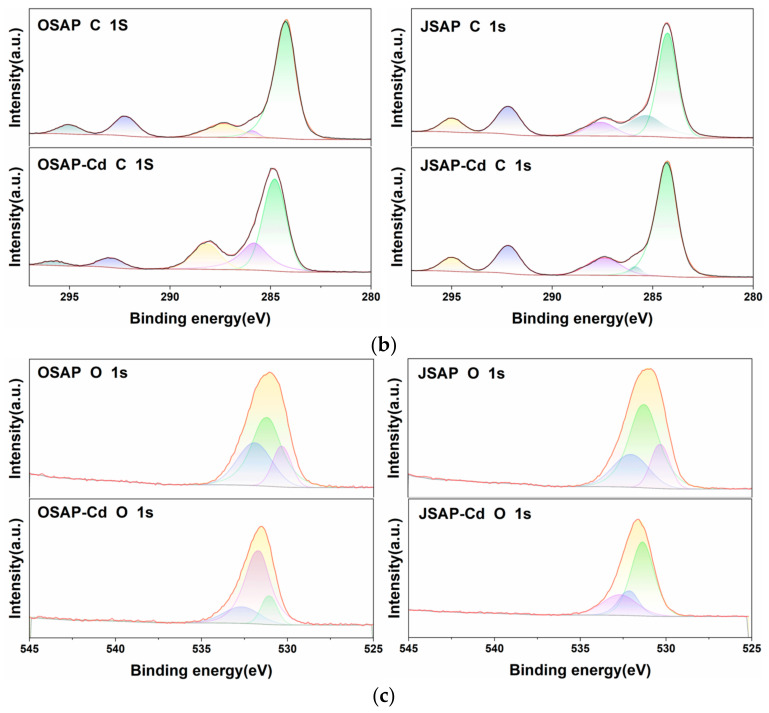
(**a**) The XPS survey spectra, (**b**) C 1s XPS spectra, and (**c**) O 1s XPS spectra of OSAP and JSAP before and after Cd(II) adsorption.

Figure 9 shows the XPS spectra of OSAP/JSAP before and after Cd(II) adsorption. One color represents one functional group, highlighting changes in functional groups before and after the reaction. As shown in Figure 9a, the appearance of a Cd(II) peak after the adsorption of Cd(II) indicates that OSAP/JSAP successfully adsorbed Cd(II). The C 1s and O 1s peaks were detected at 299.35 and 546.35 eV, respectively. Using the area simulation curve, the element O values of OSAP/JSAP surfaces were computed to be 9.82% and 11.68%, respectively. These values indicate that the surface of organic JSAP has a high oxygen content. The main elements in the O 1s spectrum are -OH (531.8 eV) and C-O (533.72 eV). The peak areas of JSAP/OSAP increased by 0.03% and 12.58%, respectively, after adsorbing Cd(II). The peak area of C-O decreased by 1.12% in JSAP and increased by 0.04% in OSAP. The oxygen-containing groups have a noticeable significant increasing tendency. The abundant oxygen-containing functional groups in OSAP-Cd and JSAP-Cd may be attributed to the loading of C-O and -OH on the material surface. Chen [14] investigated the use of synthetic mineral adsorbents to remove Cd(II) and Pb(II) ions from aqueous solutions. They discovered that the adsorption of Cd(II) by adsorbents caused an increase in the concentration of oxygen functional groups; Huang [16] contrasted the XPS spectra of the materials before and after Cd(II) adsorption. They discovered that the quantity of functional groups increased when the O 1s peak intensified after adsorption. This is consistent with the results of this research. This phenomenon could be attributed to the utilization of pure water containing -OH as the background solution in the adsorption experiment. The -OH is adsorbed onto the material’s surface, leading to an augmentation of the oxygen-containing functional groups on OSAP-Cd and JSAP-Cd. According to related research, samples containing more oxygen functional groups exhibit stronger electronegativity and complexation [25].

Figure 9b shows the C 1s spectra of OSAP/JSAP before and after Cd(II) adsorption. The results suggest that the main C 1s peaks are represented by the following four curves: C-O (phenols, alcohols, and ethers) between 285.9 and 286.1 eV; C=O (carbonyl) at 287.3–287.5 eV; O-C=O (carboxyl group) at 288.9–289.9 eV; π-π* at 291.0–292.5 eV. Figure 9c shows that the O 1s spectrum can be deconvolved into two components: C-O groups of 531.9–532.3 eV and C-OH group of 533.0–533.4 eV.

Table 4 shows the peak number and relative content of surface functional groups determined by XPS C 1s and O 1s spectra of OSAP and JSAP before and after adsorption of Cd(II). In Table 4, BE is the binding energy, and RI is the relative intensity. The OSAP-Cd sample exhibited a 5.13% and 2.53% decrease, respectively, in the O-C=O and π-π* groups, which were crucial in facilitating the adsorption of Cd(II). 

In contrast to JSAP, JSAP-Cd exhibited a 15.22% decrease in the C=O functional group, a critical factor in the adsorption process of Cd(II). Ion exchange and complexation were utilized to adsorb Cd(II) onto the O-C=O, π-π*, and C=O groups of OSAP/JSAP, as determined by analysis. In contrast to OSAP, OSAP-Cd exhibited a 12.58% increase in the -OH group and a 1.12% decrease in the C-O group. The C-O group demonstrated little effect on the adsorption of Cd(II). In contrast to JSAP, JSAP-Cd exhibited a 0.03% increase in the OH group and a 0.04% decrease in the C-O group. The oxygen-containing group demonstrated negligible influence on the adsorption of Cd(II).

#### 3.4.2. SEM Analysis 

**Figure 10:**
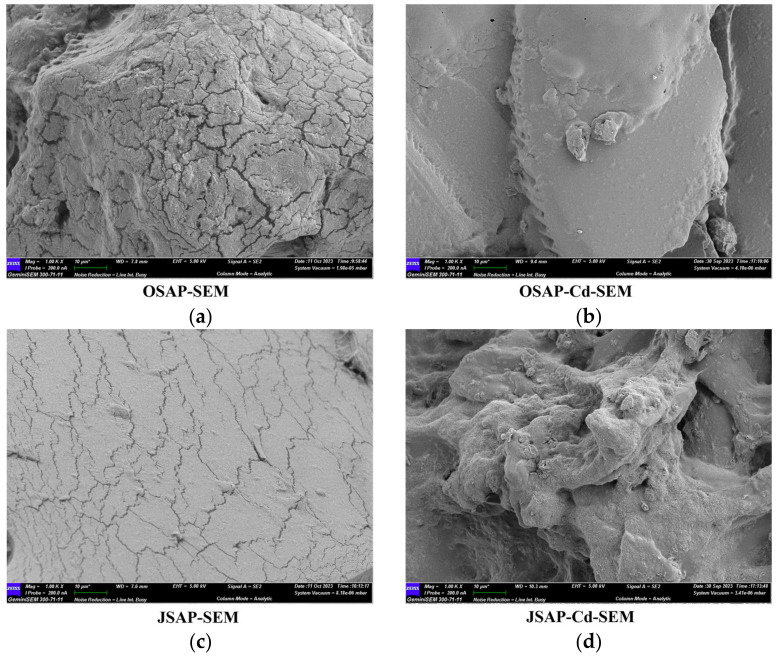
SEM micrographs. (**a**) OSAP; (**b**) OSAP-Cd; (**c**) JSAP; (**d**) JSAP-Cd.

Figure 10 shows the microscopic morphology. The surfaces of OSAP-SEM and JSAP-SEM are smooth, and cracks are caused by drying due to the lack of moisture. In the OSAP-Cd-SEM microstructure, cracks disappear, and block-like small particles appear on the surface. In the JSAP-Cd-SEM microstructure, the smooth surface becomes an irregular aggregate. Different-sized aggregates appear in both OSAP-Cd-SEM and JSAP-Cd-SEM, which may be attributed to the disruption of the SAP network structure by Cd(II). The XPS diagram also shows that Cd(II) has been successfully adsorbed on OSAP/JSAP.

#### 3.4.3. FTIR Analysis 

**Figure 11:**
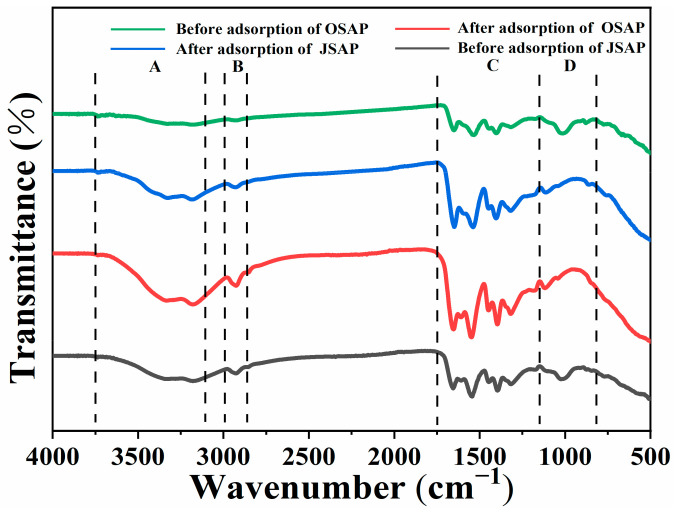
FTIR spectra of OSAP, JSAP, OSAP-Cd, and JSAP-Cd.

Figure 11 shows the FTIR spectra before and after the adsorption of Cd(II) by OSAP/JSAP. It shows that the spectra of OSAP/JSAP have been roughly categorized into four regions(A, B, C, D) based on the location of the adsorption peak: The primary part of Region A, which spans 3100–3750 cm^−1^, is hydroxyl vibrations (-OH) [26,27]; Region B, 2800–3000 cm^−1^, is the vibration region of aliphatic C-H [28]. The Region C, 1200–1750 cm^−1^, is oxygen-containing functional groups, mainly including hydroxyl, carbonyl, and carboxyl groups, as well as the stretching vibration of C=O and aromatic C=C bond structures, and the bending vibration of -CH_2_ and -CH_3_ [29]. Region D, 700–1200 cm^−1^, is unsaturated hydrocarbon C-H in-plane stretching vibration and out-of-plane deformation vibration, as well as inorganic mineral vibration. The hydroxyl adsorption peak is higher than the hydrocarbon adsorption peak and occurs at a frequency greater than 3000 cm^−1^. This phenomenon provides confirmation that -OH exists. The free -OH adsorption peak appears in the range of 3600–3700 cm^−1^, and the intramolecular association -OH adsorption peak appears in the range of 3000–3500 cm^−1^ [30]. Alcohols and phenolic hydroxyl groups appear in the range of 3000–3500 cm^−1^ wider peaks and 3600 cm^−1^ sharper peaks [31].

Before and after adsorption, the carboxylate dissociation -OH adsorption peak of OSAP/JSAP appears near 3000 cm^−1^. After the adsorption reaction, the peak value increased mildly, indicating that there was more carboxylic acid dissociation -OH. The adsorption peak of OSAP/JSAP carboxylic acid structure appeared in the range of 1400 cm^−1^. COO- in carboxylate has two C=O vibration couplings, the antisymmetric stretching vibration appeared in 1550–1610 cm^−1^, and the symmetric stretching vibration is weaker than the antisymmetric stretching vibration in 1350–1440 cm^−1^ [32]. The amine group adsorption peak of OSAP/JSAP appeared in the range of 3250 cm^−1^, the N-H bending vibration adsorption peak appeared in the range of 1550 cm^−1^, and the in-plane N-H vibration appeared in the range of 600–900 cm^−1^ [33]. The peak amino value of organic JSAP was higher than that of inorganic OSAP. The stretching vibration of the medium-strength adsorption peak of the C-N structure of OSAP/JSAP appears in the range of 1400 cm^−1^. The adsorption peak of the amide II band formed by the coupling between N-H and C-N appeared in the range of 1510–1570 cm^−1^. After the adsorption, the adsorption peak near 1400 cm^−1^ became stronger because Cd(II) reacted with the amide group to form more amide II bands. The ether adsorption peak of OSAP/JSAP appears in the range of 1020–1300 cm^−1^, that of aromatic ether in the range of 1310–1020 cm^−1^, and that of vinyl ether in the range of 1020–1075 cm^−1^ [30]. Research demonstrated that the surface hydroxyl and carboxyl groups of a substance are crucial in facilitating the binding of trace metal ions [34].

#### 3.4.4. Adsorption Mechanism

**Figure 12:**
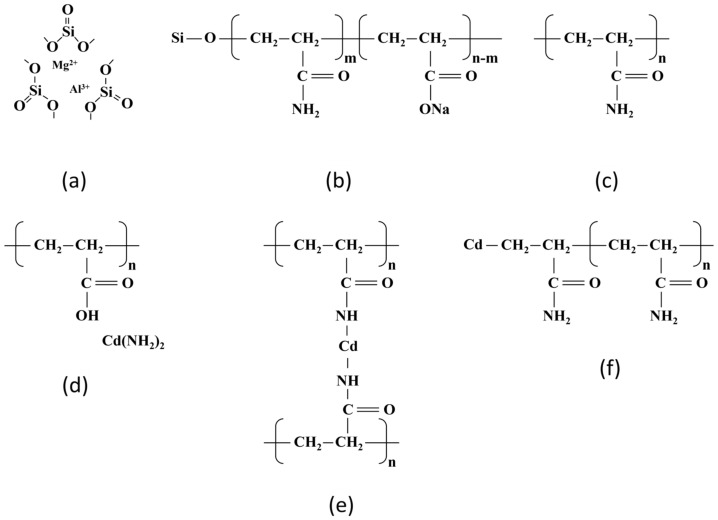
Chemical structure. (**a**) Attapulgite; (**b**) OSAP [35]; (**c**) Polyacrylamide; (**d**) Broken carbon–nitrogen bond; (**e**) Broken amide bond; (**f**) Broken carbon–carbon bond.

Figure 12 shows the chemical structural changes of the SAP before and after the chemical reaction. Figure 12a–c shows the chemical structures of attapulgite, OSAP, and JSAP, respectively. Figure 12d–f shows the potential chemical structures of polyacrylamide after the reaction. The difference between OSAP and JSAP is that OSAP introduces the silicon element and converts some of the amide groups into carboxyl groups, from a chemical structure perspective. 

Cd(II) adsorption can lead to the breakage of polymer chains through various mechanisms, including the breakage of amino groups, amide groups, and carbon–carbon bonds. Breakage of amino groups: Cd(II) may undergo hydrolysis-like reactions with the carbon–nitrogen bonds on the polyacrylamide chain, leading to chain breakage. Breakage of amide groups: In this reaction, trace metal Cd(II) coordinates with the amide groups on the polyacrylamide polymer chain, forming coordination compounds that result in chain breakage. Free radical reactions: Cd(II) may undergo free radical reactions with the carbon–carbon bonds on the polyacrylamide chain, resulting in chain breakage. This reaction may involve free radical initiators such as peroxides or heat-induced free radical generation.

Both OSAP and JSAP have the polyacrylamide chemical structure, and the chemical structural changes in this part are similar. The difference is that the Si element in OSAP suppresses free radical reactions, which prevents polymer chain breakage. Hence, OSAP possesses higher interference resistance and adsorption capacity. 

**Figure 13:**
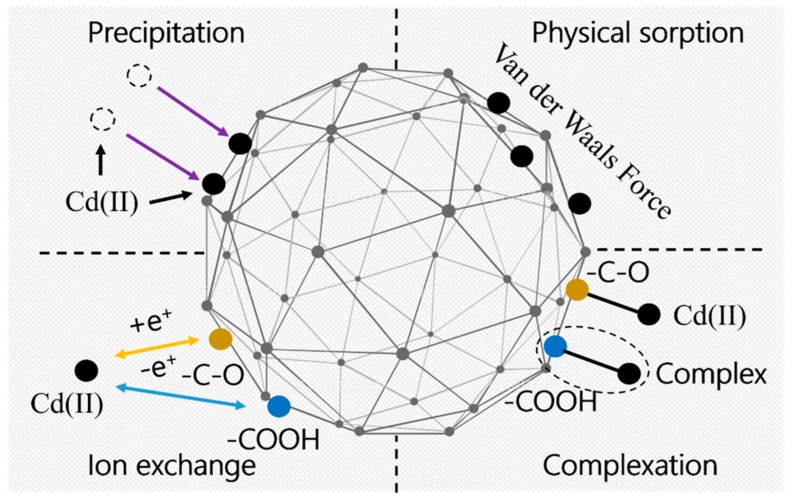
Mechanistic analysis.

Figure 13 shows the mechanism analysis of Cd(II) adsorption by SAP. Cd(II) negatively affects the water adsorption capacity of SAP. An increase in Cd(II) ion concentration reduces the water adsorption rate of SAP. The structure of the SAP usually has a polymer chain or network structure, which provides a large number of adsorption sites. The occupation of some adsorption sites by Cd(II) can lead to the disruption of the network structure of SAP. In terms of water retention, OSAP is less affected by Cd(II) compared to JSAP. OSAP is an organic–inorganic type of SAP, which is less influenced by pH relative to JSAP, making it advantageous for use in dry acidic soil environments. In terms of functional groups and structures, carboxyl (-COOH) and hydroxyl (-OH) functional groups play a key role in the adsorption of Cd(II).

The main aspects of adsorption mechanism of Cd(II) are as follows. Ion exchange: The carboxyl group (-COOH), hydroxyl group (-OH), and other functional groups in the SAP can adsorb Cd(II) through ion exchange. These functional groups ionize in solution, producing negatively charged ions. After the exchange between the negatively charged ions and the positively charged Cd(II) ions, Cd(II) is adsorbed onto SAP. Complexation: The carboxyl group and hydroxyl group in the SAP can form a stable five or six-membered ring structure complex with Cd(II), enhancing the adsorption ability. Physical adsorption: Physical adsorption mainly depends on the polymer chains or network structure of SAP, which immobilizes Cd(II) at adsorption sites through physical effects such as van der Waals forces and hydrogen bonding. Physical precipitation: The physical precipitation method mainly includes the adsorption of materials with high surface area or surface porosity. It relies on the physical properties of SAP, such as high specific surface area and porous structure, to achieve the adsorption of Cd(II).

In conclusion, the adsorption mechanism of Cd(II) by SAP is composed of multiple aspects. OSAP demonstrates advantages in arid and semi-arid environmental conditions due to its stability and acid resistance in practical applications. The research progress on adsorption of Cd(II) by SAP is shown in Table 5.

### 3.5. Effect of Cadmium Ion on Water Adsorption Characteristics of Superabsorbent Polymer

**Figure 14:**
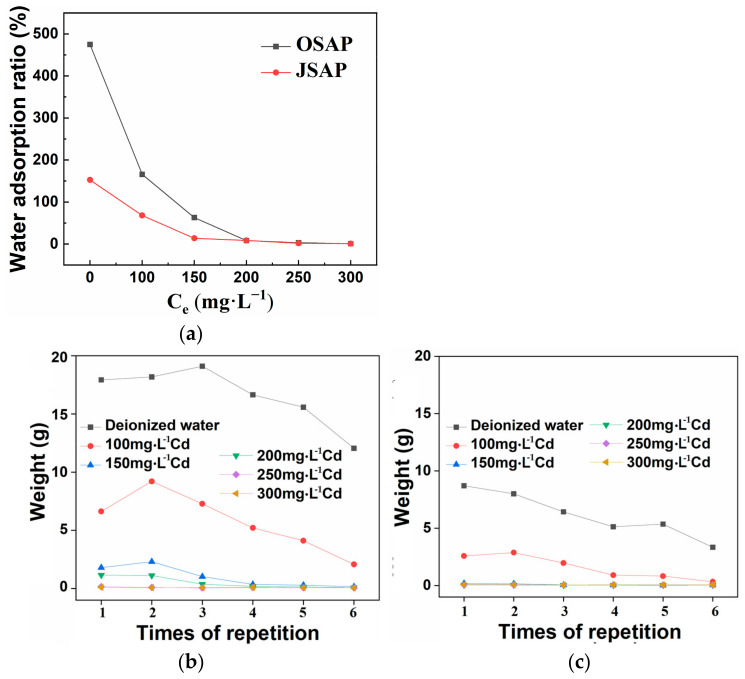

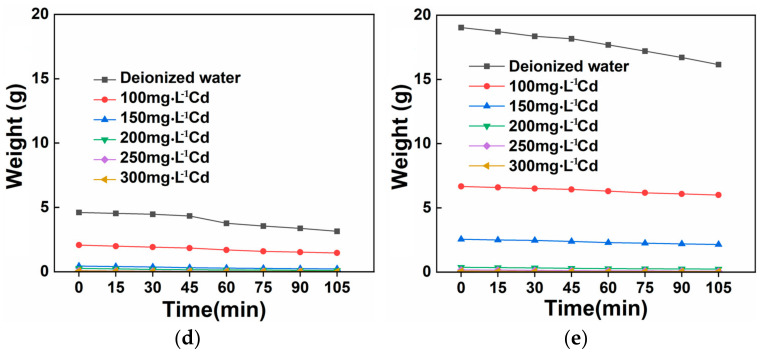
(**a**) Water adsorption ratio at different Cd concentrations. Repeated water adsorption of (**b**) OSAP and (**c**) JSAP. Water-retaining property of (**d**) OSAP and (**e**) JSAP.

As shown in Figure 14a, the water adsorption ratios of OSAP and JSAP are 474.8 and 152.6 in pure water, respectively. This provides the reference values for the adsorption capacities of SAP without Cd(II) interference. When the concentration of Cd(II) solution increases, there is a noticeable decrease in the adsorption of water by OSAP and JSAP. This indicates that Cd(II) has a negative impact on the water adsorption capacity of SAP. The water adsorption rates of OSAP and JSAP exceed 10% of that in pure water, when the concentrations of Cd(II) solution are of 150 mg·L^−1^ and 100 mg·L^−1^, respectively. This implies that, although Cd(II) has a negative impact on the water adsorption capacity of SAP, SAP can still maintain good water adsorption capacity within a certain concentration range. See Text S6 for detailed calculations.

As shown in Figure 14b,c, the water adsorption capacity of OSAP/JSAP begins to decrease with an increase in the number of repeated water adsorption cycles. In the first three cycles, the water adsorption of OSAP did not show a significant decrease. However, over the subsequent three cycles, the water adsorption of OSAP steadily decreased. Meanwhile, the water adsorption of JSAP steadily decreased over the six cycles. This suggests that the repeated water adsorption cycles affected the water adsorption capacity of SAP, whether pure water or the solution containing trace metal Cd(II). After six water adsorption cycles, OSAP and JSAP both demonstrate good repeated water adsorption performance. This could be attributed to changes in the internal structure and properties of SAP during the water adsorption process. The organic–inorganic hybrid structure of OSAP may be relatively more stable compared to the pure organic structure of JSAP.

As shown in Figure 14d,e, the weight of water adsorbed by OSAP and JSAP generally decreases over time. The rate of decline in the weight of water adsorbed over time remains relatively stable. Due to the relatively constant water release rate of SAP, the process of water release is relatively stable. The rate of decline in the weight of water adsorbed over time remains relatively stable. Due to the relatively constant water release rate of SAP, the process of water release is relatively stable.

According to the agricultural SAP monitoring standards, special attention should be given to the results after OSAP and JSAP undergo five repeated water adsorption cycles. After undergoing five cycles, both OSAP and JSAP are capable of maintaining a certain weight of adsorbed water, showcasing their capability for cyclic utilization. The changes in water adsorption performance of OSAP/JSAP over multiple cycles can be attributed to differences in their structure, composition, and water adsorption mechanisms.

### 3.6. Evaluation and Analysis the Economy of SAP

From the perspective of economic analysis, OSAP and JSAP have significant differences in raw material cost and finished product price, which directly affects their market competitiveness and profit potential.

Table 6 shows the price reference. First, in terms of raw material costs, OSAP has a clear advantage. Attapulgite, as the main raw material of OSAP, has a relatively low price, at only USD 41.40–69.00 per ton. The low-cost source of raw materials allows OSAP to maintain low costs in the production process, giving it a greater price advantage in the market competition. In contrast, the raw material cost of JSAP is higher, especially the key raw material of acrylamide, which costs between USD 1104.00 and 1379.00 per ton. This makes JSAP face greater cost pressure in raw material procurement, which affects its final selling price and profitability.

Secondly, from the point of view of the price of finished products, the market price of SAP on the market at present is USD 2484.00–8280.00 per ton, the current market price of OSAP is USD 1794.00–2070.00 per ton, and the price of JSAP is USD 3450.00–4140.00 per ton. JSAP per ton is more expensive than OSAP, mainly due to JSAP using higher-cost acrylamide raw materials. In summary, from the economic perspective, OSAP is more suitable for the remediation of trace metal cadmium pollution in arid and semi-arid agricultural areas. The price for OSAP and JSAP can be found on the official website.

## 4. Conclusions

This research investigated the influence of Cd(II) on the adsorption and water absorption characteristics of OSAP and JSAP. pH was identified as the primary factor affecting Cd adsorption. The adsorption behavior of both OSAP and JSAP is spontaneous and exothermic. The main adsorption mechanisms of OSAP and JSAP are precipitation, physical sorption, ion exchange, and complexation. In ion exchange, the participation of H_2_O and abundant OH^−^ groups play a significant role in Cd(II) adsorption. OSAP demonstrates better performance compared to JSAP in terms of acid resistance, maximum adsorption capacity, stability, and water absorption. Due to its lower-cost raw materials, OSAP exhibits a cost advantage over JSAP.

These results provide theoretical support for the selection of different types of water-retaining agents and greatly complement the understanding of the interaction between two types of SAPs and Cd(II). The organic–inorganic OSAP is suitable for agricultural production in arid and semi-arid regions and ecological restoration of cadmium-contaminated soil.

## Figures and Tables

**Table 1:** Langmuir, Freundlich, and Temkin isotherm constants for Cd(II) adsorption.

Isotherm	Parameters	Sample	
		OSAP	JSAP
Langmuir		$Q_{e}=\frac{Q_{m}K_{L}C_{e}}{1+K_{L}C_{e}}$	
	Q_m_/(mg·g^−1^)	770.037 ± 74.317	345.369 ± 43.306
	K_L_/(L·mg^−1^)	0.002	0.006
	R^2^	0.977–0.997	0.966–0.996
Freundlich		$Q_{e}=K_{F}{C_{e}}^{1/n}$	
	K_F_/((mg·g^−1^)(L·mg^−1^)·n^−1^)	3.426 ± 0.592	10.560 ± 1.754
	n	1.295	1.862
	R^2^	0.970–0.970	0.935–0.935
Temkin		$Q_{e}=\frac{RT}{b}InC_{e}+\frac{RT}{b}InK_{T}$	
	b/(KJ·mol^−1^)	59.38	31.89
	K_T_/(L·mg^−1^)	5.75	3.53
	R^2^	0.774–0.774	0.949–0.949

**Table 2:** Thermodynamic parameters on OSAP and JSAP.

Adsorbent	Intercept	Slope	Temp (K)	$∆G^{{}^{\circ}}$	$∆H^{{}^{\circ}}$	$∆S^{{}^{\circ}}$	$R^{2}$
OSAP	3.226 ± 0.035	−0.042 ± 0.001	288.15	−7.728	0.349	26.821	0.998–0.999
			298.15	−7.996			
			308.15	−8.264			
JSAP	2.525 ± 0.004	−0.020 ± 5.275	288.15	−6.048	0.166	20.993	0.999–0.999
			298.15	−6.258			
			308.15	−6.468			

**Table 3:** Parameters of kinetics models and intra-particle diffusion models for the adsorption of Cd(II) onto OSAP and JSAP.

Kinetic Models	Parameters	Samples	
		OSAP	JSAP
Pseudo-first-order parameters	Q_e_/(mg·g^−1^)	210.114 ± 2.599	159.109 ± 2.368
	K_1_/(min^−1^)	2.436	2.117
	R_1_^2^	0.302–0.380	0.369–0.439
Pseudo-second-order parameters	Q_e_/(mg·g^−1^)	213.480 ± 2.034	162.486 ± 1.708
	K_2_/(g·mg^−1^·min^−1^)	0.033	0.031
	R_2_^2^	0.684–0.719	0.761–0.787

**Table 4:** Peak numbers and relative content of the surface functional groups determined by C 1s and O 1s spectra from XPS for OSAP and JSAP before and after Cd(II) adsorption.

Element	Functional Groups	OSAP		JSAP		OSAP-Cd		JSAP-Cd	
		BE/eV	RI/%	BE/eV	RI/%	BE/eV	RI/%	BE/eV	RI/%
C 1s	C-C	284.18	55.63	284.25	37.53	284.79	39.09	284.24	48.76
	C-O	285.91	1.72	285.29	17.38	285.83	17.14	285.87	2.16
	C=O	287.39	9.22	287.59	7.01	288.13	12.95	287.43	10.54
	O-C=O	292.19	9.47	292.19	11.9	292.94	4.34	292.18	12.14
	π-π*	295.03	4.39	294.99	5.64	295.76	1.86	294.99	5.72
O 1s	C-O	531.18	9.82	531.26	11.68	531.06	3.5	531.26	11.76
	C-OH	530.34	3.32	530.33	3.59	531.67	15.9	530.33	3.62
	C-O	531.93	6.43	532.06	5.27	532.67	5.22	532.06	5.31

**Table 5:** Current research progress on the effect and conditions of adsorption of cadmium ions by SAP.

Name of the Adsorbent	pH	Initial Cd(II) Concentration (mg·L^−1^)	Max Adsorption Capacity (mg·g^−1^)	Reaction Conditions	Reference
Microwave synthesized guar gum-graft-poly (ethylacrylate)	9.0	100	714.28	Temperature = 30 ± 2 °C Contact time = 16 h Dose = 50 g	https://doi.org/10.1021/ie801416z (accessed on 10 June 2024) [36]
The hydrous manganese oxide poly (acrylamide-co-sodiumacrylate) (PPM)	6.0	100	698	Temperature = 25 °C Contact time = 2 h Dose = 20 mg	https://doi.org/10.1016/j.matdes.2016.02.025 (accessed on 10 June 2024) [37]
Poly(acrylamide-co-sodium acrylate) (PP)	6.0	100	281	Temperature = 25 °C Contact time = 2 h Dose = 20 mg	https://doi.org/10.1016/j.matdes.2016.02.025 (accessed on 10 June 2024) [37]
Conventionally synthesized guar gum-graft-poly (ethylacrylate)	9.0	100	270.27	Temperature = 30 ± 2 °C Contact time = 16 h Dose = 50 g	https://doi.org/10.1021/ie801416z (accessed on 10 June 2024) [36]
Poly[4-(4-vinylbenzyloxy)-2-hydrobenzaldehyde] (PVBH)	5.5	0.1–2.0	250	–	https://doi.org/10.1016/j.jhazmat.2009.05.028 (accessed on 10 June 2024) [38]
Polyacrylamide-grafted iron(III) oxide	6.0	50–200	147.2	Temperature = 30 ± 8 °C Dose = 0.2 g·L^−1^	https://doi.org/10.1016/S0304-3894(01)00392-2 (accessed on 10 June 2024) [39]
OSAP/JSAP	5.5	100	770/345	This research	This research

**Table 6:** Price reference.

Name	Price per Ton	Reference Links
Attapulgite	USD 41.40–69.00	https://doi.org/10.1016/j.cej.2023.141404 https://www.made-in-china.com/products-search/hot-china-products/Attapulgite_Clay.html URL (1 June 2024)
Acrylamide	USD 1104.00–1379.00	https://www.made-in-china.com/products-search/hot-china-products/Acrylamide.html URL (1 June 2024)
SAP	USD 2484.00–8280.00	https://www.made-in-china.com/products-search/hot-china-products/Superabsorbentpolmer.html URL (1 June 2024)
OSAP	USD 1794.00–2070.00	http://show.guidechem.com/lhk1981/ URL (1 June 2024)
JSAP	USD 3450.00–4140.00	http://watersap.com/ URL (1 June 2024)

## Data Availability

The data are unavailable due to privacy.

## Supplementary Materials

The following supporting information can be downloaded at: https://www.mdpi.com/article/10.3390/polym16121756/s1, Text S1: the adsorption capacity of Cd(II); Text S2: Adsorption isotherms experiment of materials for Cd(Ⅱ); Text S3: Kinetic adsorption experiment of materials for Cd(Ⅱ); Text S4: Desorption quantity and desorption rate calculation; Text S5: ΔG_0_, ΔH_0_ and ΔS_0_ calculation; Text S6: Water absorption ratio. References [40,41,42] are cited in the Supplementary Materials.
