# Supplementary material for: Study on Adsorption Characteristics and Water Retention Properties of Attapulgite–Sodium Polyacrylate and Polyacrylamide to Trace Metal Cadmium Ion
Source: Polymers (Basel). 2024 Jun 20;16(12):1756. doi: 10.3390/polym16121756 (PMC11207512; doi:10.3390/polym16121756)
Supplement: Supplementary file 1 [file polymers-16-01756-s001.zip › polymers-3023155-supplementary.pdf]

Supplementary Material

Study on Adsorption Characteristics and Water Retention Properties of Attapulgite–Sodium Polyacrylate and Polyacrylamide to Trace Metal Cadmium Ion

Ziming Cai [1], Feng Zhan [1,*] Yingnan WANG [2], Meiling Wu [2], Lingjian Kong [2], An Wang [2] and Zhanbin Huang [2,*]

[1] School of Resources, Environment and Materials, Guangxi University, Nanning 530004, China; 2015391002@st.gxu.edu.cn

[2] School of Chemical and Environmental Engineering, China University of Mining and Technology (Beijing), Beijing 100083, China; wangyingsouth@163.com (Y.W.); zqt2100302085@cumtb.edu.cn (M.W.); ljkong2023@163.com (L.K.); wanganchina@126.com (A.W.)

* Correspondence: phy_idea@outlook.com (F.Z.); zbhuang202208@126.com (Z.H.)

Text S1 the adsorption capacity of Cd(II):

$$Q_e = \frac{(C_0 - C_e)V}{m}$$

where Q_e (mg·g⁻¹) is the amount of Cd(II) adsorbed, C_0 (mg·g⁻¹) and C_e (mg·g⁻¹) are the concentrations of Cd(II) at the initial and equilibrium concentration, respectively. V (ml) presents the volume of the suspension, and m (mg) represents the mass of the adsorbent.

Text S2 Adsorption isotherms experiment of materials for Cd(Ⅱ)

The Langmuir isotherm model [1]:

$$Q_e = \frac{Q_m K_L C_e}{1 + K_L C_e}$$

The Freundlich isotherm model [2]:

$$Q_e = K_F {C_e}^{1/n}$$

The Temkin equation [3]:

$$Q_e = \frac{RT}{b} InK_T + \frac{RT}{b} InC_e$$

where Q_e (mg·g^{-1}) is the amount of Cd(II) adsorbed at equilibrium, C_e (mg·g^{-1}) represents the concentration of Cd(II) at the adsorption equilibrium, Q_m (mg·g^{-1}) is the maximum sorption capacity and K_L (L·mg^{-1}) is the Langmuir constant; K_F ((mg·g^{-1})(L·mg^{-1})·n^{-1}) and n are the Freundlich constants referring to sorption capacity and intensity, respectively. T and R are the absolute temperature (K) and the universal gas constant (8.314 J·mol^{-1}·K^{-1}), K_T is the Temkin maximum binding energy isotherm constant in L (mg^{-1}), b is the Temkin constant.

Text S3 Kinetic adsorption experiment of materials for Cd(Ⅱ)

The pseudo-first-order kinetic model is described as follows:

$$Q_t = Q_e(1 - e^{-k_1 t})$$

The pseudo-second-order kinetic model is described as follows:

$$Q_t = \frac{k_2 {Q_e}^2 t}{1 + k_2 Q_e t}$$

where Q_e (mg·g^{-1}) is the amount of Cd(II) adsorbed at equilibrium, Q_e (mg· g^{-1}) is the amount of Cd(II) adsorbed on different time t (min), k_1 (min^{-1})and k_2 (g·mg^{-1}·min^{-1})are the rate constants of two kinetic models, respectively. Adsorption pH envelopes, adsorption isotherms, and kinetics adsorption experiments were conducted in this Cd(II) adsorption experiments.

Text S4 Desorption quantity and desorption rate calculation

The desorption quantity calculation is described as follows:

$$D_e = \frac{C_e \times V}{m}$$

where D_e (mg·g^{-1}) is the desorption quantity of the adsorbent for Cd(II), C_e (mg·g^{-1}) is the equilibrium concentration of Cd(II) in the solution after desorption, V (ml) is the volume of the solution, and m (mg) is the amount of adsorbent added.

The desorption rate calculation is described as follows:

$$Desorption\ rate = \frac{D_e}{Q_e}$$

where D_e (mg·g^{-1}) is the desorption quantity of the adsorbent for Cd(II), Q_e (mg·g^{-1}) is the amount of Cd(II) adsorbed.

Text S5 ΔG_0, ΔH_0 and ΔS_0 calculation

The ΔG_0, ΔH_0 and ΔS_0 calculation is described as follows:

$$\Delta G_0 = -RTIn|K|$$

$$\Delta G_0 = \Delta H_0 - T\Delta S_0$$

where K is the equilibrium constant. In a two-dimensional graph with the vertical axis as $In(\frac{Q_e}{C_e})$ and the horizontal axis as C_e, the slope is K. R is the gas constant (8.314 J·mol^{-1}·K^{-1}). T is absolute temperature (K).

Text S6 Water absorption ratio

The dried OSAP and JSAP are placed in distilled water until their weight stabilizes. Then, they are removed from the water and weighed. The calculation formula for swelling ratio is as follows：

$$Swelling\ ratio = \frac{Ws - Wd}{Wd}$$

where Wd and Ws is the weight of the dried sample and the sample at swelling equilibrium state, respectively.

References

1. Johnson, R.D.; Arnold, F.H., The temkin isotherm describes heterogeneous protein adsorption. *Biochim. Biophys. Acta-Protein Struct. Molec. Enzym.* **1995**, *1247*, 293–297.
2. Wang, F.T.; Pan, Y.F.; Cai, P.X.; Guo, T.X.; Xiao, H.N. Single and binary adsorption of heavy metal ions from aqueous solutions using sugarcane cellulose-based adsorbent. *Bioresour. Technol.* **2017**, *241*, 482–490.
3. Allen, S.J.; McKay, G.; Porter, J.F. Adsorption isotherm models for basic dye adsorption by peat in single and binary component systems. *J. Colloid Interface Sci.* **2004**, *280*, 322–333.
